# Can vegetation be discretely classified in species‐poor environments? Testing plant community concepts for vegetation monitoring on sub‐Antarctic Marion Island

**DOI:** 10.1002/ece3.9681

**Published:** 2023-01-03

**Authors:** Stephni van der Merwe, Michelle Greve, Andrew Luke Skowno, Michael Timm Hoffman, Michael Denis Cramer

**Affiliations:** ^1^ Department of Biological Sciences University of Cape Town Cape Town South Africa; ^2^ Kirstenbosch Research Centre South African National Biodiversity Institute Cape Town South Africa; ^3^ Department of Plant and Soil Sciences University of Pretoria Pretoria South Africa

**Keywords:** cluster analysis, discrete vegetation, plant community, species‐poor environments, sub‐Antarctic Marion Island, vegetation classification

## Abstract

The updating and rethinking of vegetation classifications is important for ecosystem monitoring in a rapidly changing world, where the distribution of vegetation is changing. The general assumption that discrete and persistent plant communities exist that can be monitored efficiently, is rarely tested before undertaking a classification. Marion Island (MI) is comprised of species‐poor vegetation undergoing rapid environmental change. It presents a unique opportunity to test the ability to discretely classify species‐poor vegetation with recently developed objective classification techniques and relate it to previous classifications. We classified vascular species data of 476 plots sampled across MI, using Ward hierarchical clustering, divisive analysis clustering, non‐hierarchical kmeans and partitioning around medoids. Internal cluster validation was performed using silhouette widths, Dunn index, connectivity of clusters and gap statistic. Indicator species analyses were also conducted on the best performing clustering methods. We evaluated the outputs against previously classified units. Ward clustering performed the best, with the highest average silhouette width and Dunn index, as well as the lowest connectivity. The number of clusters differed amongst the clustering methods, but most validation measures, including for Ward clustering, indicated that two and three clusters are the best fit for the data. However, all classification methods produced weakly separated, highly connected clusters with low compactness and low fidelity and specificity to clusters. There was no particularly robust and effective classification outcome that could group plots into previously suggested vegetation units based on species composition alone. The relatively recent age (*c.* 450,000 years B.P.), glaciation history (last glacial maximum 34,500 years B.P.) and isolation of the sub‐Antarctic islands may have hindered the development of strong vascular plant species assemblages with discrete boundaries. Discrete classification at the community‐level using species composition may not be suitable in such species‐poor environments. Species‐level, rather than community‐level, monitoring may thus be more appropriate in species‐poor environments, aligning with continuum theory rather than community theory.

## Graphical abstract

We reexamined the classification of vegetation on sub‐Antarctic Marion Island and found that there were no strong floristic grounds for a discrete classification. A community‐level classification using species composition may not be suitable in such species‐poor environments. Species‐level, rather than community‐level, monitoring may be more appropriate in species‐poor environments.

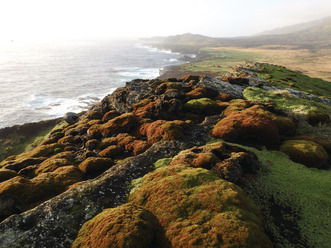

## 1 INTRODUCTION

Plant ecologists identify, describe and map vegetation variation that represents underlying ecological processes in an effort to understand the complex spatial and temporal interactions between taxa and the environments in which they occur (De Cáceres et al., 2015). While vegetation variation is complicated and arguably varies along a continuum in space and time, humans tend to think categorically. This requires simplification through classification to create useful, logical and manageable units for theoretical and practical purposes (De Cáceres et al., 2015; Wiser & De Cáceres, 2013). The aim of vegetation classification is to delineate and describe environments using the characteristics of the standing vegetation (De Cáceres et al., 2015) to provide a surrogate for ecosystem delineation (Brown et al., 2013). A vegetation classification serves as baseline data for ecosystem research, land‐use planning, environmental assessments and scientifically based decisions in biodiversity management (Brown & Bredenkamp, 2018). Policy‐making, conservation and research therefore depend on accurate and up to date description and delineation of vegetation units.

Plant ecology concepts have evolved over time, and have recently enjoyed renewed interest, especially in terms of updating and advancing previous classifications (De Cáceres et al., 2015; Mucina et al., 2016; van Staden et al., 2021). Early approaches to vegetation variation viewed vegetation as either hierarchical, compositionally distinct units (“communities”) that vary as an entire unit in space and time (i.e., discrete community concept; Weaver & Clements, 1929) or entities made up of a continuum of a temporary co‐occurrence of species that fluctuate in composition, space or time (i.e., the continuum concept; Curtis & McIntosh, 1951). The continuum concept proposes that vegetation does not consist of homogeneous persistent units, but is the outcome of individual species' responses to their environment and to each other (Palmer & White, 1994). This concept is related to the niche concept which proposes that each species partitions a resource along a gradient (Austin, 2013). The two extremes in approaches viewed vegetation as either a super‐organism of co‐evolved groups of species (i.e., community) or as species that assemble entirely individualistically (Austin, 2013). No consensus has been reached on which perspective is most appropriate for classifying particular environments (Austin, 2013; Austin & Smith, 1989; Curtis & McIntosh, 1951; Lortie et al., 2004; Scott, 1995; Weaver & Clements, 1929). However, the categorical, compositionally discontinuous, discrete model of plant communities, initially proposed by Weaver and Clements (1929), persists mainly due to historical legacy and its utility in creating vegetation maps for ecological management (Feilhauer et al., 2020). While the two approaches are not necessarily incompatible, most ecologists interested in vegetation description continue to define vegetation as an assemblage of distinct hierarchical plant communities (De Cáceres et al., 2018; Gremmen, 1981; Mucina et al., 2016; Tsakalos et al., 2018; van Staden et al., 2021).

The general assumption of the community concept, that discrete and persistent vegetation units exist, is rarely tested before undertaking a classification, with the exception of more recent research (Feilhauer et al., 2020; Lortie et al., 2004; Pavão et al., 2019). This raises concerns about the widespread use of the traditional community concept and the application of methods developed that have underlying assumptions rooted in the existence of homogenous discrete spatial entities. Assuming, a priori, that specific floristically distinct communities exist may disregard the unique vegetation patterns often found in environments with few vascular plant species such as in Aquatic (Landucci et al., 2015) or Tundra (Yang et al., 2021) vegetation. In recent decades, a variety of new tools have been developed for vegetation scientists (see, e.g., Aho et al., 2008 or Lötter et al., 2013). While these approaches may encourage new perspectives on the complex nature of vegetation patterns, they bring new challenges, for example, in the selection of appropriate clustering methods (Maechler et al., 2019; Oksanen et al., 2020). Lötter et al. (2013) referred to this as “the classification conundrum”. The amount of research available which advocates particular methods, ideologies and approaches to classify vegetation (Feilhauer et al., 2020; Lengyel et al., 2021; Lortie et al., 2004; Lötter et al., 2013; Pakgohar et al., 2021), reflects the impracticality of the use of one universal approach in all environments. Nevertheless, there is general agreement that expert opinion is needed to select vegetation units at some stage in the classification process (Brown et al., 2013; Lötter et al., 2013; Mucina, 1997) even if this adds subjectivity to the classification, possibly resulting in bias (Lötter et al., 2013; Wolda, 1981), with little objective validation of clustering results. However, recent classification methods, especially those used in data science (Flynt & Dean, 2016), have made it possible to formally test the effectiveness of classifications, thereby reducing the number of subjective choices (Lötter et al., 2013; Pakgohar et al., 2021). The existence of discrete groups in the data can thus be tested objectively, before expert interpretation is needed.

Updating and rethinking vegetation classification is especially important in tracking shifts in the distribution of species in response to changes in climate and other anthropogenic drivers. In the sub‐Antarctic, the regional climate has changed at an accelerated pace compared with lower latitudes (le Roux & McGeoch, 2008a). For example, between 1949 and 2003, Marion Island (MI) has experienced an increase in mean annual temperature from 5.4 to 6.4°C, which is double the mean global rate of increase (le Roux & McGeoch, 2008a). Mean annual rainfall has also decreased from *c*. 3000 mm to *c*. 2000 mm during the same period (le Roux & McGeoch, 2008b). The vegetation is closely coupled with abiotic conditions and consists of 23 native vascular plant species and ranges from near continuous short‐statured plant cover in sub‐Antarctic Tundra to barren Polar Desert (Smith & Mucina, 2006). The island is remote, has a relatively recent origin—only emerging above sea level for the first time *c*. 450,000 B.P. (McDougall et al., 2001)—and has been glaciated with the greatest extent of ice occurring most recently *c*. 34,500 years ago during the last glacial maximum (Rudolph et al., 2020). Rapid climatic change has already altered the distribution and relationships between plant species and perhaps redistributed some species which were used to previously classify communities on MI (le Roux & McGeoch, 2008b). In addition, a more temperate climate coupled with anthropogenic disturbances has created new opportunities for the establishment and spread of non‐native species (Greve et al., 2017). Three alien plant species have become widespread on MI (*Poa annua*, *Sagina procumbens*, *Cerastium fontanum*), especially in areas influenced by animals near the coast (le Roux et al., 2013). The most widespread invasive species on MI is the House Mouse (*Mus musculus*) which has rapidly increased in density, abundance and distribution since feral cats, originally introduced to control mice, were eradicated in 1991 (McClelland et al., 2018). The mice impact most aspects of the biodiversity of MI including causing mortality in plant species (Phiri et al., 2009), decreasing invertebrate abundance (Smith et al., 2002), increasing seabird deaths (Dilley et al., 2016) and reducing indigenous seed caches (Smith et al., 2002). Due to both invasive species and climate change impacts, the vegetation has been changing on MI and is expected to change significantly in the near‐future with the planned mouse eradication (Preston et al., 2019), with monitoring becoming a key conservation objective for the island.

To effectively study and monitor the impact of climate change and alien species on the vegetation, an ecologically meaningful vegetation classification and monitoring unit is needed that can be objectively and repeatedly defined, mapped and monitored at a fine scale. Since using remote sensing data for image classification of vegetation in the near permanent cloud cover experienced on most islands in the sub‐Antarctic is challenging (Fitzgerald et al., 2021), a floristic approach to classification using plot data may show intrinsic vegetation patterns and thus act as a proxy for underlying environmental variation and patterns that form the standing vegetation. Using a floristic classification may uncouple the vegetation distribution from previous assumptions of environmental drivers and allow modeling of change in abiotic conditions with resultant groups acting as the units of change.

Here, we tested whether vegetation forms compositionally discrete units in a species‐poor environment, which are generally neglected environments in classification research. MI presents an opportunity to test plant community concepts and to elucidate appropriate classification approaches in species‐poor environments that are closely coupled with abiotic conditions. The first objective was to identify and differentiate vegetation units using both hierarchical and non‐hierarchical classification algorithms. The second objective was to compare and validate clustering methods. The third objective was to describe the vegetation units using indicator species analysis (ISA). The best performing clustering method was related to earlier classifications that used phytosociological relevè table sorting based on vascular and bryophyte species (Gremmen, 1981) and a cluster analysis using scores from an ordination based largely on soil chemistry and plant guilds (Smith et al., 2001) to classify vegetation. Since previous research suggested viewing the vegetation as discontinuous, we expected compositionally well separated vegetation groups where the variation between groups can be related to abiotic and biotic influences.

## 2 METHODS

### 2.1 Study site

**FIGURE 1:**
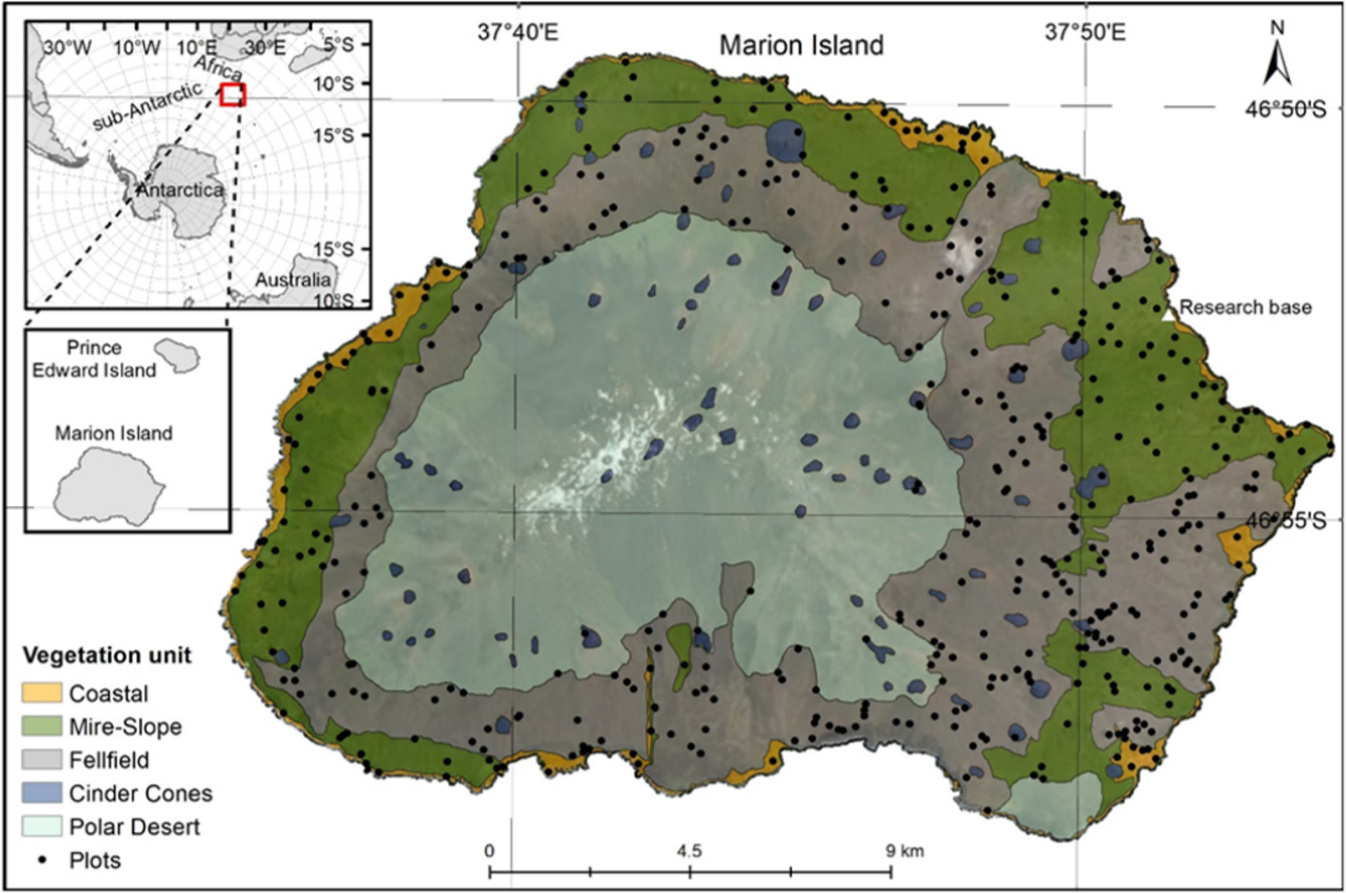
Vegetation map of MI showing the five units (in color) delineated by Smith and Mucina (2006). Black points indicate the location of plots sampled in this study.

Marion Island (46°54′S, 37°45′E) is a volcanic, remote, sub‐Antarctic island covering an area of *c*. 290 km^2^ (Figure 1). The South African‐governed island has a cool, thermally‐stable, oceanic climate with mean annual precipitation of *c*. 2000 mm (le Roux & McGeoch, 2008a). The islands' geology consists of smoothed pre‐glacial gray lava and rough post‐glacial black lava (McDougall et al., 2001) with *c*. 130 more recent red scoriaceous cinder cones scattered around the island (Rudolph et al., 2020; Figure 1). The vegetation changes along an elevational severity gradient (le Roux & McGeoch, 2008c), from the coast to the highest elevation of 1230 m, and generally occurs in two layers at lower elevations. These are a prostrate vascular plant layer, rarely exceeding 50 cm in height and a low ground cover of bryophytes (Gremmen, 1981). The flora comprises 23 indigenous species (Chau et al., 2020) and 17 alien vascular plant species (Greve et al., 2017), along with 134 bryophyte and 100 lichen species (Øvstedal & Gremmen, 2001). Many alien vascular plant species have been controlled and only occur in isolated locations (Greve et al., 2017).

Five vegetation units have been mapped previously based on field research, photographs and field observations, and informed by expert opinion (Smith & Mucina, 2006; Figure 1). Smith and Mucina (2006) recognized that mapping at the scale of plant community identified in previous studies (Gremmen, 1981; Smith & Steenkamp, 2001), in vegetation that changes within a few meters, would not be possible, and thus mapped five units (Figure 1) at a broader scale. Polar Desert was indicated by the absence of vascular plant species and by the presence of bryophytes (Smith & Steenkamp, 2001). Cinder cones, conspicuous red volcanic ash deposits, were largely associated with bryophytes, although Gremmen (1981) included cinder cones under Fellfield vegetation. Fellfield is dominated by *Azorella selago* cushion plants and epiphytic *Polypogon magellanicus* grasses, with several vascular plant species co‐occurring at lower altitudes (Smith & Steenkamp, 2001). The Mire‐Slope unit is made up of the Mire and Slope communities combined, as mapping at the fine scale needed to differentiate Mires and Slopes was not possible (Smith & Mucina, 2006). Slope communities are either dominated by the fern *Austroblechnum penna‐marina* or shrub *Acaena magellanica* (on slopes with impeded drainage). Mires occur on flat or slightly sloping areas, dominated by graminoids *P. magellanicus* and *Uncinia compacta* and various bryophytes. Lastly, Coastal vegetation is either largely dominated by *Crassula moschata* (exposed to high salt spray) or by *Liptinella plumosa*, *Callitriche antarctica* or *Poa cookii* (in areas influenced by biotic activity; Smith & Steenkamp, 2001).

### 2.2 Vegetation sampling

Vegetation data consisted of 476 vegetation plots that were sampled on MI using systematic randomized sampling in 2018 and 2019. Plot locations thus included a wide range of environmental conditions (Figure 1). In each 3 × 3 m plot, the percentage ocular canopy cover of all vascular plant species was estimated by trained observers following Daubenmire (1959). The percentage cover of two non‐vegetated cover classes were also estimated: bare rock or soil and open water. A description of the vegetation was produced for each plot in the field to assist the classification. Two bryophytes were identified to genus level, namely *Breutelia* and *Brachythesium*, and three to species‐level namely *Marchantia polymorpha*, *Marchantia berteroana* and *Racomitrium lanuginosum*. These bryophytes were easily identified in field and were indicator species for plant communities in previous classifications (Gremmen, 1981). All other bryophytes were estimated collectively as “bryophytes”. Lichens were also given a collective cover estimate. To reduce noise, species with two or less observations in the matrix were removed and thus rare species were not considered (e.g., Addicott et al., 2018). Five alien species were recorded in the data, with only *Poa annua*, *Sagina procumbens* and *Cerastium fontanum* retained in analyses after rare species were removed. All analyses were initially conducted on two subsets of the data: including versus excluding the three alien species. However, the optimality of clustering did not improve with their exclusion, and these alien species were thus included in the analyses. Indeed, Smith et al. (2001) suggested that alien species should be included in classifications due to the increasingly important role of invasive species on community function, structure, and dynamics.

### 2.3 Cluster analysis

To select the most robust classification procedure for our study site, the best practice was to test a variety of procedures to determine if the vegetation data do indeed form clusters that can be interpreted ecologically (Aho et al., 2008; Lötter et al., 2013). The classifications were undertaken in three steps: (1) pre‐processing involved the selection of a distance measure and normalization of the data; (2) cluster analysis involved the selection and application of the clustering algorithm and its various parameters; (3) cluster validation involved the selection and application of appropriate internal validation techniques to evaluate the quality of the classification. Four clustering algorithms and four validation measures were explored based on demonstrated performance in recent literature (Aho et al., 2008; Handl et al., 2005; Lengyel et al., 2021; Pakgohar et al., 2021). We defined a vegetation classification as being comprised of a cluster of plots organized into units with discrete boundaries between them. The aim was to identify clusters of plots containing small within‐cluster variance (i.e., compact clusters) and sufficiently large between‐cluster variance (i.e., spatially well‐separated). All analyses were conducted in R Statistical Software v. 4.02 (R Core Team, 2020).

One divisive and three agglomerative clustering algorithms prominent in the literature were tested using the raw data. Divisive analysis clustering (DIANA) (Maechler et al., 2019) was chosen as the divisive hierarchical clustering method; it starts with all plot data in one cluster and successively divides plots based on a “distance” metric, selected by the researcher, into clusters. Conversely, agglomerative hierarchical clustering starts with each plot as an individual cluster locating pairs of plots with the smallest distance, fusing the two plots into a cluster. The approach then re‐iteratively calculates the distance from fused plots to all remaining plots until all sites are grouped into one cluster. For agglomerative clustering, the hierarchical Ward clustering method was chosen after comparison to single, average and complete linkage clustering (linkage refers to the way the distance measure is implemented to form clusters; see Aho et al., 2008 for a summary of the linkage methods). This was done by calculating the agglomerative coefficient and divisive coefficient for DIANA in the “cluster” package in R (Maechler et al., 2019). The Ward method aims to minimize the within‐cluster variance and searches for clusters in multivariate Euclidean space (Murtagh & Legendre, 2014). The Ward Method, which showed the strongest clustering, implements squared Euclidian distances based on sum of squares (Murtagh & Legendre, 2014), but is not appropriate for non‐metric distance (e.g., Bray‐Curtis), thus Euclidean distance was chosen as the dissimilarity metric, calculated using the “vegan” package (Oksanen et al., 2020). To include non‐hierarchical classification, kmeans and partitioning around medoids (PAM) clustering were chosen as centroid‐based algorithms that identify *k* centroids, allocating each data point to the nearest centroid. Kmeans aims to minimize the sum of squared distances of data points to their cluster centroid, whereas PAM minimizes dissimilarity between data points in a cluster and its cluster centre (medoids). Initial investigations showed that all dissimilarity measures explored (i.e., Hellinger, Manhattan and Bray‐Curtis distances) with single, average and complete linkage, where possible, produced similar results (see also Aho et al., 2008).

### 2.4 Number of clusters

There is no consensus on an ideal measure to estimate the optimum number of clusters or most appropriate clustering method (Aho et al., 2008; Lötter et al., 2013). To choose the optimum number of clusters for each clustering method, we used (1) silhouette widths, (2) Dunn index and the (3) gap statistic in the “NbClust” package (Charrad et al., 2014). Silhouette width is widely used to simultaneously determine the optimum number of clusters and quality of the entire classification (Handl et al., 2005). Silhouette width estimates the average distance between clusters, i.e., how close data points in a cluster are to data points in neighboring clusters (Rousseeuw, 1987). The Dunn index calculates the ratio between maximum intra‐cluster distance and minimum inter‐cluster distance (Dunn, 1974). The gap statistic compares within‐cluster distance to a uniformly distributed null reference distribution with bootstrapping (Tibshirani et al., 2001). The optimum cluster number is indicated where the gap curve reaches an inflection point and changes to a higher value. Previous classifications of the vegetation on MI defined between five and 41 vegetation units (Gremmen, 1981; Huntley, 1971; Smith et al., 2001; Smith & Mucina, 2006), so there was no a priori reason to choose any particular number of clusters. However, we explored five clusters along with the optimal number of clusters indicated by the validation measures, to compare to the suggested five vegetation units mapped previously (Smith & Mucina, 2006).

### 2.5 Cluster validation

Since various R packages have been created for internal cluster validation, multiple packages and validation measures were explored. We evaluated optimality as maximizing intra‐cluster homogeneity and inter‐cluster distance, and minimizing the degree to which a cluster groups data points together with the nearest neighbors (Handl et al., 2005). To determine the optimal clustering method based on compactness, separation and connectivity (the three most important clustering criteria) of each clustering algorithm, the (1) silhouette widths, (2) Dunn index and (3) connectivity of clusters were calculated for two to 20 clusters with the “clValid” package (Brock et al., 2008). Individual silhouette plots were drawn for each clustering method with the “cluster” package (Maechler et al., 2019) using the optimal number of clusters per method. These plots show the silhouette widths estimated for each plot within a cluster and calculates the average silhouette width (ASW) for each cluster. Both the Dunn index and silhouette width compute a final score that combines two clustering criteria: compactness and separation (Handl et al., 2005). Connectivity indicates the degree to which clusters are connected to the nearest neighbors to determine to what extent data items are placed in the same cluster as their nearest neighbor (Saha & Bandyopadhyay, 2012). While most of these are heuristic methods, well‐separated and compact clusters are indicated by large silhouette widths and Dunn index values. Ideally, connectivity should be minimized so that plots nearby are more related than plots further away. Furthermore, dendrograms and centroids assisted to visually determine groupings in the data.

#### 2.5.1 Indicator species analysis

Indicator species analysis (ISA) was conducted with the “indicspecies” package (De Cáceres & Legendre, 2009), to determine the association of diagnostic species with each cluster and to compare with previously classified groups which were indicated by particular species (Gremmen, 1981). The analysis was run for the optimal number of clusters in the best performing clustering method and also for five clusters, to compare to the five groups that were previously proposed in the vegetation map (Smith & Mucina, 2006). The ISA is based on an Indicator Value (Dufrêne & Legendre, 1997) that calculates a plant species' relative abundance and frequency of occurrence to estimate the strength of species associations with the predetermined groups (Dufrêne & Legendre, 1997). The statistical significance of the association is then tested with a permutation test (De Cáceres & Legendre, 2009). This analysis thus indicates species fidelity (the probability of finding the species in plots that belong to the cluster) and specificity (the probability that a plot belongs to the cluster given that the species is present in the plot). Fidelity is fundamental to interpreting the association of species with a vegetation group under the Braun‐Blanquette approach. These two components combined gives an association statistic to a group.

A vegetation map was created with the output of the classification using ArcGIS Desktop© and Google Earth Pro©. We used Google Earth satellite imagery, the previous vegetation map (Smith & Mucina, 2006), plot data from 2018 to 2020 and a digital surface model of the island to inform the map.

## 3 RESULTS

### 3.1 Cluster analysis

**FIGURE 2:**
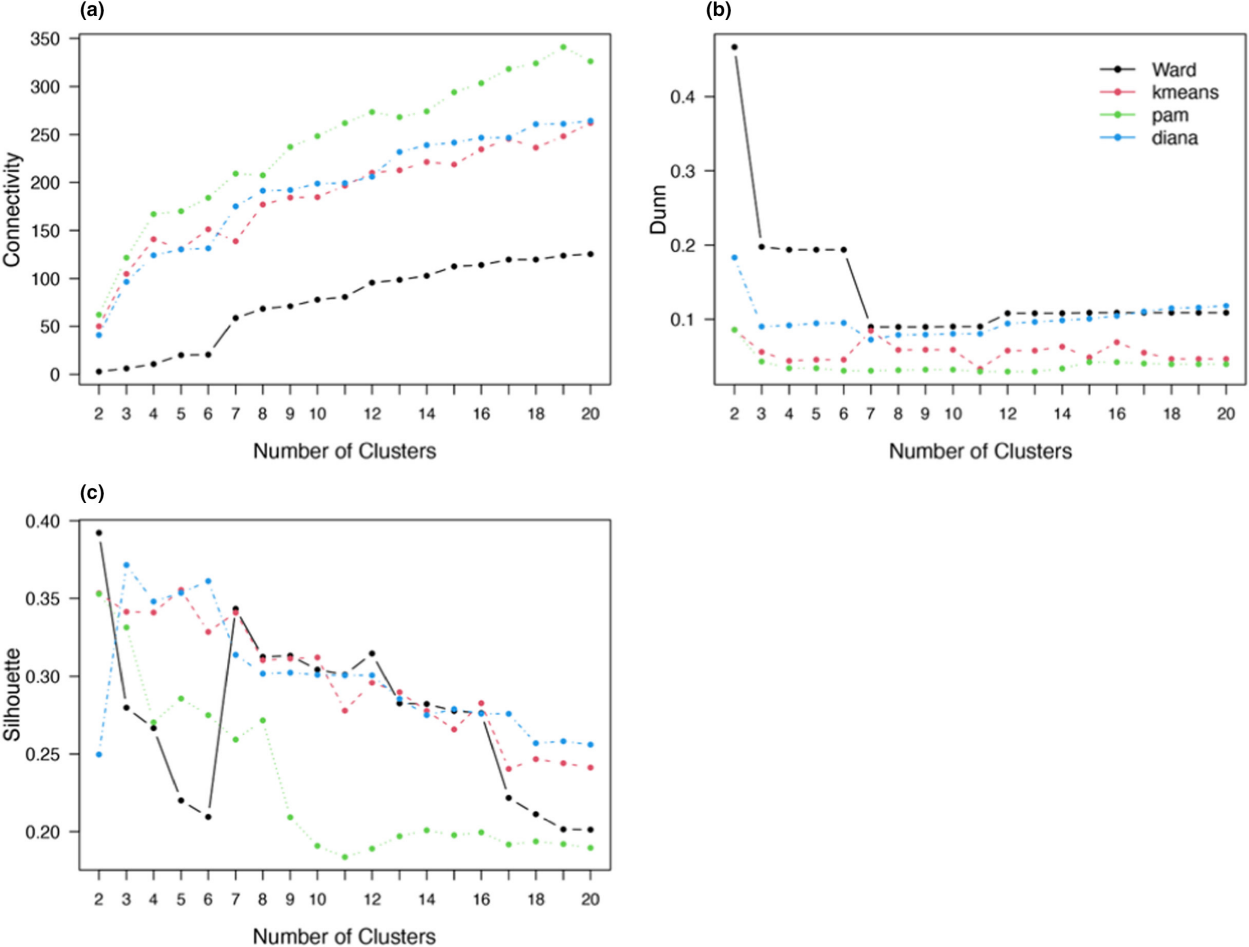
Cluster validation measures to determine the optimal clustering method between Ward, kmeans, PAM and DIANA clustering (indicated by the colors) using (a) connectivity, (b) Dunn index, and (c) ASW for two to 20 clusters. The Dunn index calculates the ratio between maximum intra‐cluster distance and minimum inter‐cluster distance, and ASW estimates the average distance between clusters. Large Dunn index and silhouette width values thus indicate compact and well separated vegetation groups. Connectivity refers to the connectivity of clusters to nearest neighbors and should ideally be low so that plots nearby are more related than those further away.

Ward hierarchical clustering consistently performed better than kmeans, DIANA and PAM clustering in all validation measures (Figure 2). Ward clustering also had the highest agglomerative coefficient (0.98), compared with single (0.80), complete (0.89) and average (0.86) linkage, and the divisive coefficient for DIANA (0.87). Ward clustering had the highest ASW (0.39, Table 1) and Dunn index (0.47, Table 1). It also had the lowest connectivity for any number of clusters (Figure 2).

**TABLE 1:** Cluster validation results of the four cluster analyses

Method	ASW	Dunn	Connectivity	Rank
Ward	0.39	0.47	2.93	1
DIANA	0.37	0.18	40.94	2
kmeans	0.36	0.086	50.02	3
PAM	0.35	0.086	62.05	4

*Note*: Only the highest values for ASW and Dunn index, as well as lowest values for connectivity are shown. “Rank” indicates the best to worst performing clustering method based on all three validation measures.

### 3.2 Number of clusters

**FIGURE 3:**
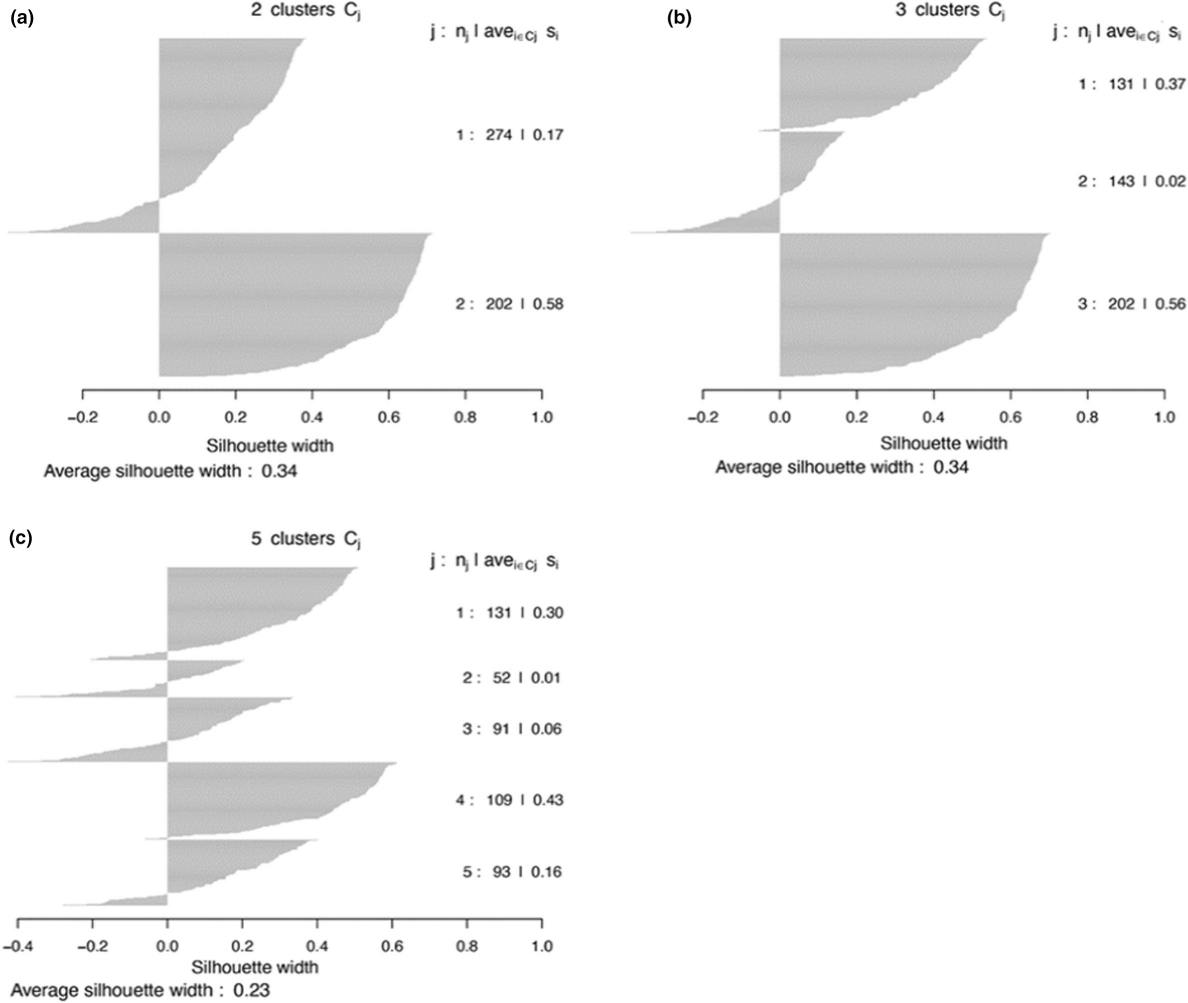
Silhouette plots of Ward hierarchical clustering for (a) two, (b) three and (c) five clusters. The number of clusters (*n* = 2 and 3) were chosen based on the two clustering solutions with highest ASW and Dunn statistic. Five clusters were chosen based on the number of vegetation complexes suggested by Smith and Mucina (2006) for MI. Each gray horizontal line represents the silhouette width of a plot that was allocated to each cluster (*j*). The number of plots (*n*_*j*_; *n* = 476) allocated to each cluster and the ASW for each cluster (ave_ieCiSi_) is shown on the right, as well as the overall average of the entire classification (shown below the graph). Small within‐cluster ASW values indicate that plots within a cluster are compositionally dissimilar. A small overall ASW for the entire classification indicates that clusters are not well separated and compact. Negative silhouette values indicate plots might have been placed in the incorrect cluster. Ideally, the plots clustered within a group would all have high and similar silhouette widths, i.e., the gray lines would be uniform within a cluster. The overall average would also ideally be high in a well separated and compact grouping of a data set.

**FIGURE 4:**
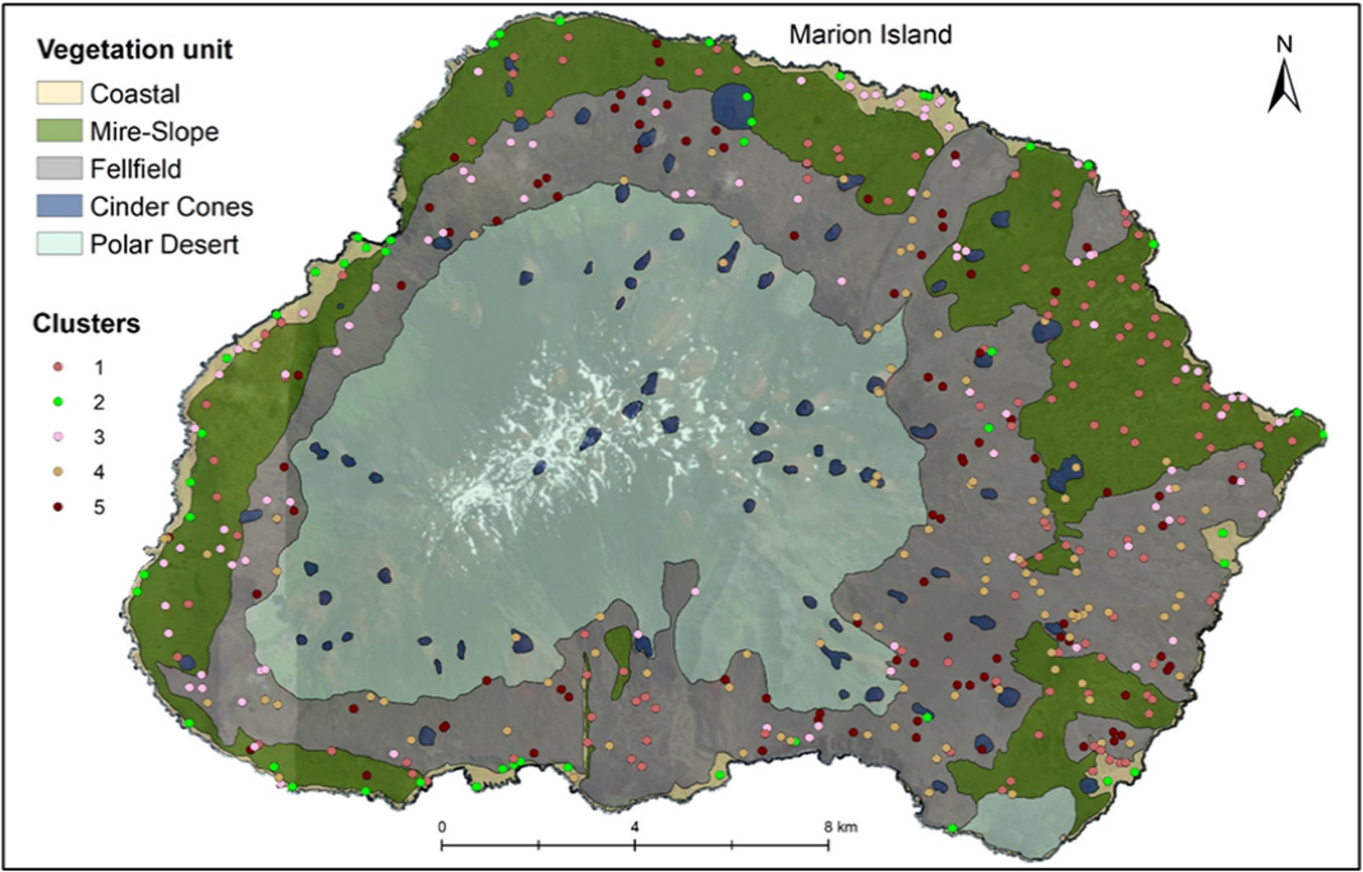
Smith and Mucina's (2006) vegetation map of MI (same as in Figure 1) with the location of plots allocated to each cluster in this study indicated as five differently‐colored dots. The five clusters are the result of the Ward cluster analysis with five groups, chosen to compare to the five mapped vegetation units. The five clusters do not match well with the five vegetation units.

**FIGURE 5:**
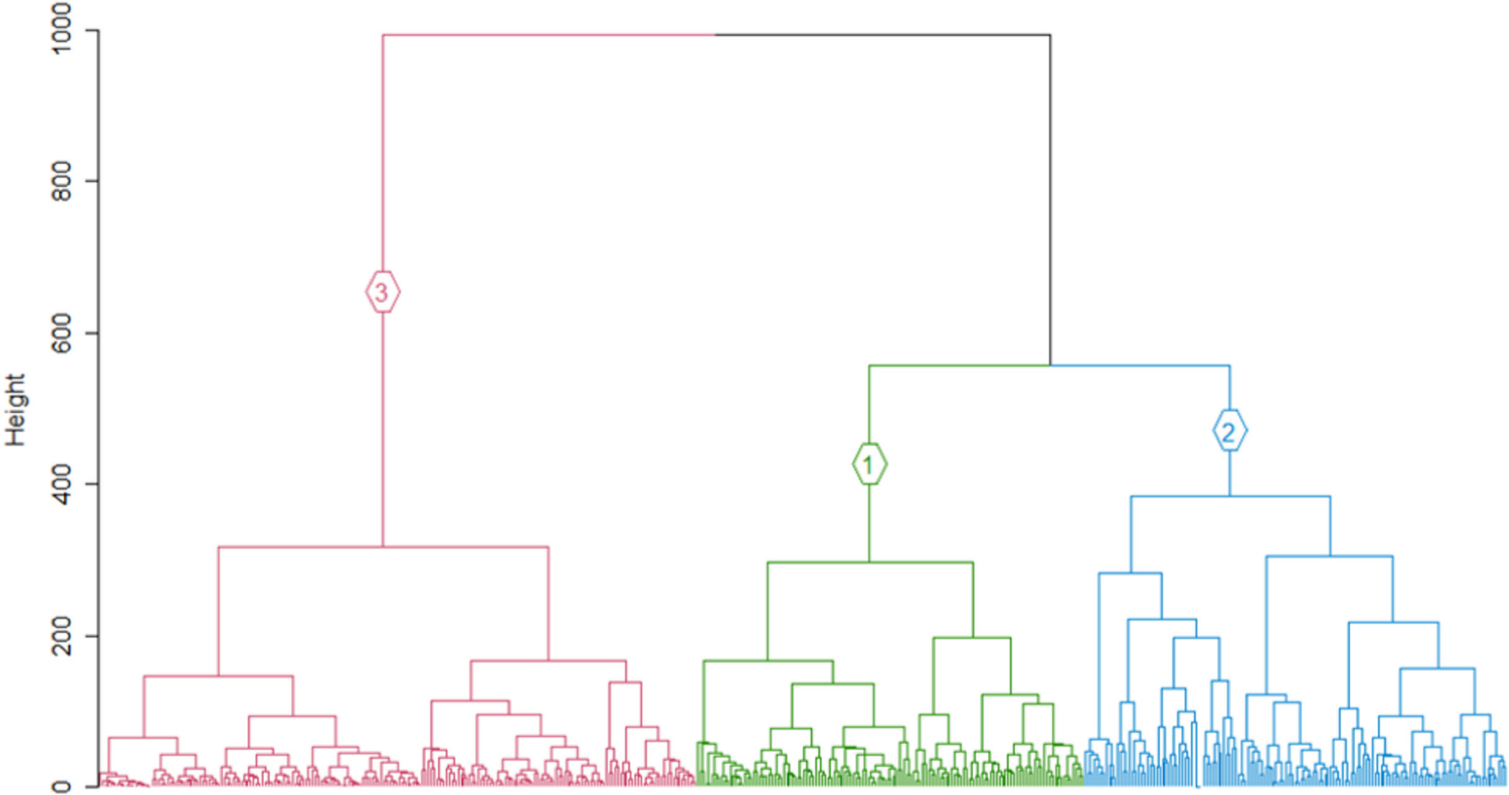
Ward hierarchical clustering dendrogram showing three clusters (colored). Height indicates the Euclidian distance between clusters and the horizontal end points show the 476 plots. Short distances (i.e., small differences in height) between data points indicate similarity. Below height = 400 or three clusters, branches split at relatively short distances, indicating low separation between clusters or high similarity. The height of the link that joins two clusters is the longest.

In all methods, clustering performance decreased with increasing cluster number (Figure 2). Most validation measures indicated that two clusters are the best fit for the data (Table 2). The ASW indicated that the data were clustered most strongly when the Ward method was clustered in two (0.34) or three (0.34) groups (Figure 3). DIANA had the highest ASW for three (0.37) and seven clusters (0.36), followed by kmeans with the highest ASW for two (0.36) and three (0.35) clusters (Table 2). PAM clustering had the highest ASW for two (0.35) and three (0.33) clusters (see Figures A1–A3 in the Appendix S1 for detailed results). The Dunn index was the highest in the two‐cluster solution for all clustering methods, decreasing with the number of clusters (Figure 2). If the first inflection point of the gap curve is considered, two clusters are suggested for Ward, kmeans and PAM, and three clusters for DIANA (see Figures A4–A7 in the Appendix S1 for detailed results). None of the validation methods indicated five groups as a good fit for the data (Figure 4). The highest linkage distance of the Ward cluster dendrograms also visually indicate two or three clusters may be appropriate for the data (Figure 5), as below three clusters (Height = 400), the linkage distance is short (i.e., the groups are not well separated; Figure 5). The Ward method with two, three and five clusters was chosen for the ISA.

**TABLE 2:** Summary of the suggested number of clusters for each validation method for the four cluster analysis methods

Method	ASW	Dunn	Gap
Ward	2	2	2
DIANA	3	2	3
kmeans	2	2	2
PAM	2	2	2

*Note*: See Figures A1–A7 in the Appendix S1 for detailed results.

### 3.3 Cluster validation

The presence of clusters with below average silhouette scores, as well as the fluctuation in the thickness of silhouette width group sizes in the Ward two, three and five cluster solutions (Figure 3), indicate suboptimal groupings for the data. Based on the overall silhouette width and Dunn index ranges of all clustering methods, which generally increase with cluster compactness and separation, most clustering methods produced weak separation, low compactness, and high connectivity (Figure 2). None of the algorithms produced strong clusters, as even the highest ASW was still low (Table 1). Centroids for kmeans and PAM clustering are also visually not well separated for neither two nor five clusters (see Figures A8–A11 in the Appendix S1 for further detail). Therefore, overall, there is no strong clustering tendency in the data.

The Ward clustering with five groups does not spatially match the previously mapped units (Figure 4). The low overall ASW for the five‐cluster solution also indicates poor clustering of groups (Figure 3). The in‐field descriptions of vegetation in plots confirmed that plots were not classified correctly, according to previously suggested units.

### 3.4 Indicator species analysis

If two clusters are selected (Table 3), *A. penna‐marina*, *Brachythesium*, *P. cookii*, *L. plumosa*, *Juncus scheuchzerioides*, *M. polymorpha*, *P. annua* and *C. antarctica* are indicator species for cluster 1 (i.e., there is high specificity for these species). Furthermore, *A. magellanica*, *U. compacta* and *C. moschata* are significant (*p* < .05) indicators species for cluster 1 based on high specificity, but are not strongly associated to the cluster (Table 3). None of the species occur in all or most plots belonging to cluster 1 (i.e., there is low fidelity). Lichen and *Notogrammitis crassior* are indicator species for cluster 2 (Table 3).

**TABLE 3:** ISA results showing species that are associated with two vegetation groups

Species	Cluster	Specificity	Fidelity	Association statistic	*p* value
*Austroblechnum penna‐marina*	1	0.93	0.69	0.80	.001
*Acaena magellanica*	1	0.89	0.52	0.68	.001
*Uncinia compacta*	1	0.83	0.34	0.53	.001
*Brachythesium spp*.	1	0.97	0.19	0.43	.001
*Poa cookii*	1	0.97	0.30	0.53	.001
*Leptinella plumosa*	1	0.98	0.13	0.35	.001
*Juncus scheuchzerioides*	1	0.94	0.11	0.32	.001
*Marchantia polymorpha*	1	1.0	0.10	0.30	.001
*Poa annua*	1	1.0	0.06	0.24	.001
*Crassula moschata*	1	0.84	0.06	0.21	.05
*Callitriche antarctica*	1	0.98	0.04	0.19	.02
Lichen	2	0.87	0.69	0.78	.001
*Notogrammitis crassior*	2	0.90	0.10	0.30	.002

*Note*: Only results for significant (*p* < .05) indicator species are shown. The ISA is based on a species' relative abundance and frequency of occurrence to estimate the strength of species associations within the predetermined groups. Specificity indicates the probability that the plots belong to the group given that the species has been found. Fidelity estimates the probability of finding the species in the plots belonging to the group. These two components combined give an association statistic. Strong indicator species would have fidelity and/or specificity values close to 1.

If three clusters are selected (Figure 5; Table 4), *A. penna‐marina* is a good indicator for cluster 1; it occurs in almost all plots belonging to this cluster (i.e., high fidelity), and is largely restricted to cluster 1 (Table 4)*. Leptinella plumosa*, *M. polymorpha*, *Montia fontana*, *P. annua*, *C. antarctica*, *C. moschata*, open water and *Collobanthus kergeulensis* are good indicators for cluster 2 (Table 4), with almost all plots containing these species belonging to cluster 2 (i.e., high specificity), although they do not occur in all plots belonging to the cluster. Furthermore, most plots that contain *P. cookii* and *Sagina procumbens* also belong to this cluster (Table 4). No species occurs in all plots that belong to cluster 2 (Table 4). Lichen appears in many plots belonging to cluster 3 and is mostly restricted to cluster 3. *Notogrammitis crassior* is also a good indicator species for cluster 3 with most plots containing this species belonging to cluster 3 (Table 4).

**TABLE 4:** ISA results showing significant indicator species for three vegetation clusters

Species	Cluster	Specificity	Fidelity	Association statistic	*p* value
*Austroblechnum penna‐marina*	1	0.87	0.99	0.93	.001
*Poa cookii*	2	0.85	0.35	0.55	.001
Water	2	0.94	0.06	0.23	.002
*Collobanthus kergeulensis*	2	0.97	0.03	0.17	.03
*Leptinella plumosa*	2	0.99	0.24	0.49	.001
*Marchantia polymorpha*	2	0.99	0.15	0.39	.001
*Sagina procumbens*	2	0.81	0.19	0.39	.002
*Poa annua*	2	0.98	0.10	0.32	.001
*Crassula moschata*	2	0.91	0.10	0.31	.05
*Callitriche antarctica*	2	0.99	0.07	0.26	.03
*Montia fontana*	2	0.98	0.13	0.35	.002
Lichen	3	0.77	0.69	0.73	.001
*Notogrammitis crassior*	3	0.82	0.10	0.29	.001
*Acaena magellanica*	1 + 2	0.94	0.52	0.70	.001
*Uncinia compacta*	1 + 2	0.91	0.34	0.56	.001
*Brachythecium*	1 + 2	0.99	0.19	0.43	.001
*Juncus scheuchzerioides*	1 + 2	0.97	0.11	0.33	.001
*Racomitrium*	1 + 3	0.90	0.62	0.75	.001
*Lycopodium magellanicum*	1 + 3	0.98	0.08	0.28	.02
*Ranunculus biternatus*	2 + 3	0.85	0.22	0.43	.003

*Note*: The ISA is based on a plant species' relative abundance and frequency of occurrence to estimate the strength of species associations with the predetermined clusters (De Cáceres & Legendre, 2009). Specificity indicates the probability that the plots belong to the cluster given that the species has been found. Fidelity estimates the probability of finding the species in the plots belonging to the cluster. These two components combined give an association statistic to a cluster. Strong indicator species would have fidelity and/or specificity values close to 1. Only results for significant (*p* < .05) indicator species are shown.

**FIGURE 6:**
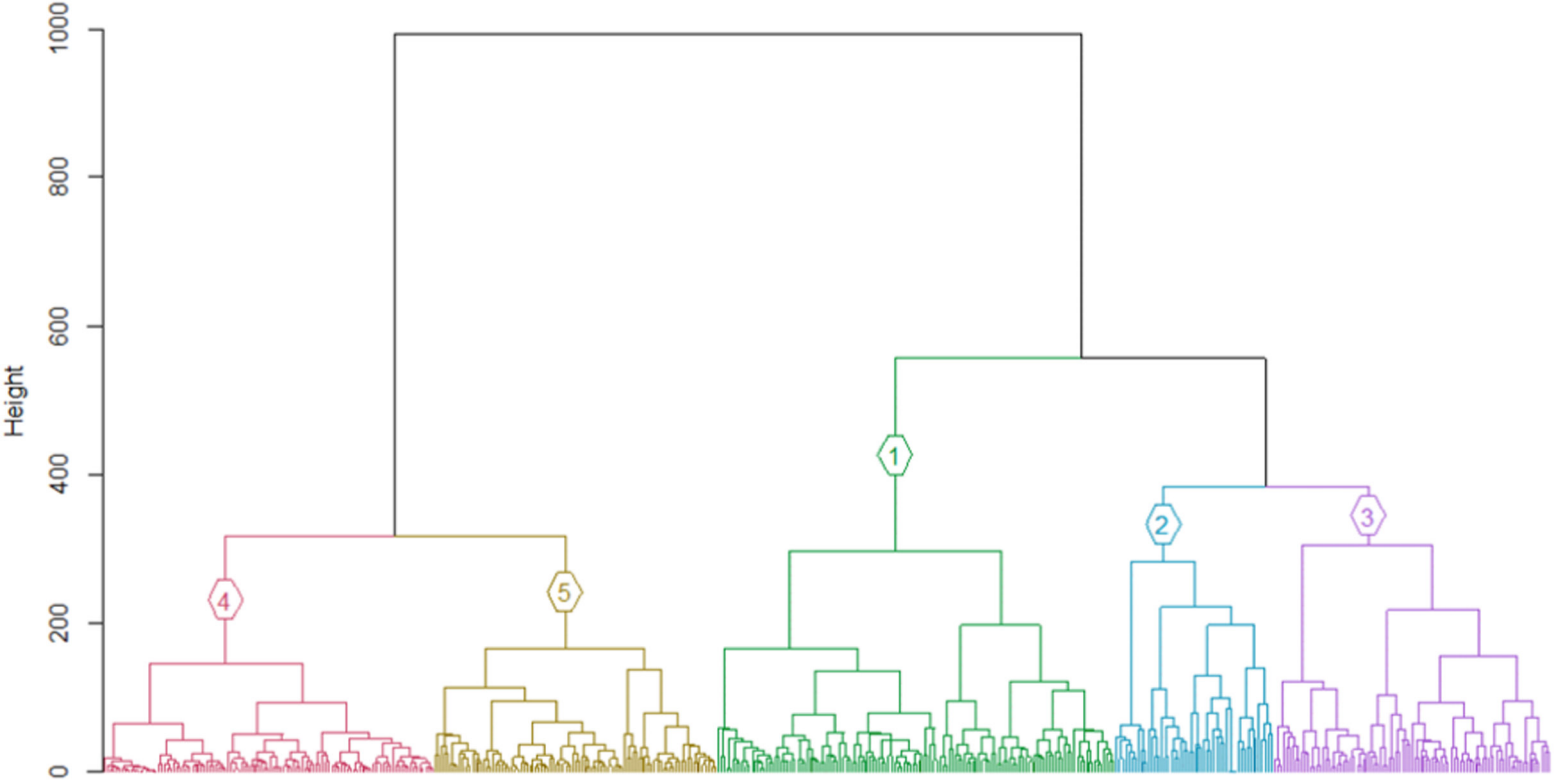
Ward hierarchical clustering dendrogram showing five clusters (colored). Height indicates the Euclidian distance between clusters and the horizontal end points show the 476 plots. Short distances (i.e., small differences in height) between data points indicate similarity. Below height = 400 or three clusters, branches split at relatively short distances, indicating low separation between clusters or high similarity. The height (*c*. 350) of the links that split the dendrogram into five clusters is at a low linkage distance, indicating a small difference between clusters.

If five clusters are selected (Figure 6), *A. penna‐marina* is a good indicator for cluster 1, as it occurs in all plots belonging to this cluster (i.e., high fidelity), and it is mostly restricted to cluster 1 (Table 5). *Leptinella plumosa*, *M. fontana*, *P. annua*, *C. antarctica*, *C. kergeulensis*, *C. moschatta* were significant indicator species (*p* < .05) for cluster 2, and to a lesser extent also *P. cookii* and *M. polymorpha* (Table 5). They are good indicator species for this cluster because they mostly occur in sites belonging to this cluster only (i.e., high specificity). No species occurs in all plots in cluster 2. Cluster 5 is indicated by *N. crassior* as most plots that contain this species belong to cluster 5. Other species are indicators for a combination of vegetation clusters, but none are good indicators for only cluster 3 or cluster 4.

**TABLE 5:** ISA results showing significant indicator species for five vegetation clusters, as suggested by previous research (Smith & Mucina, 2006)

Species	Cluster	Specificity	Fidelity	Association statistic	*p* value
*Austroblechnum penna‐marina*	1	0.78	0.99	0.88	.001
*Leptinella plumosa*	2	0.97	0.52	0.71	.001
*Poa cookii*	2	0.81	0.56	0.67	.001
*Poa annua*	2	0.99	0.29	0.54	.001
*Crassula moschata*	2	0.92	0.27	0.50	.001
*Marchantia polymorpha*	2	0.86	0.25	0.46	.001
*Callitriche antarctica*	2	0.91	0.15	0.37	.001
*Collobanthus kergeulensis*	2	0.98	0.077	0.27	.001
*Notogrammitis crassior*	5	0.79	0.14	0.33	.002
*Brachythecium*	1 + 2	0.94	0.25	0.48	.001
*Uncinia compacta*	1 + 3	0.82	0.41	0.58	.001
*Montia fontana*	2 + 3	0.98	0.13	0.35	.002
Water	2 + 3	0.92	0.06	0.23	.01
Lichen	4 + 5	0.84	0.69	0.76	.001

*Note*: The ISA is based on a plant species' relative abundance and frequency of occurrence to estimate the strength of species associations with the predetermined clusters (De Cáceres & Legendre, 2009). Specificity indicates the probability that the plots belong to the cluster given that the species has been found. Fidelity estimates the probability of finding the species in the plots belonging to the cluster. These two components combined give an association statistic to a cluster. Strong indicator species would have fidelity and/or specificity values close to 1. Only results for significant (*p* < .05) indicator species are shown.

## 4 DISCUSSION

The low clustering tendency in all methods indicates that the vegetation of MI is not well differentiated by cover‐abundance. Since all classification attempts failed to strongly cluster vegetation into stable units on MI, vascular plant species may not form compositionally discrete communities based on aerial cover. In all methods, the clusters that were generated were not well isolated and were also not ecologically meaningful based on the presence of particular species suggested by the ISA. Bricher (2012) similarly tested various clustering methods and found that clusters were not well isolated for the species‐poor vegetation on the sub‐Antarctic Macquarie Island, concluding that stable groupings could not be found in the floristic data and suggesting individual species distributions rather be used to differentiate vegetation. Macquarie Island has a similar climate, age, plant functional groups and species richness (45 vascular plant species) to MI (Bricher, 2012). Therefore, this study suggests that a discrete community concept may not be appropriate for species‐poor vegetation.

Our initial intention was to update previous vegetation classifications, but the plot data could not robustly be divided into the previously mapped five vegetation units (Smith & Mucina, 2006) or into the plant communities suggested in earlier classifications (Gremmen, 1981; Huntley, 1971; Smith & Steenkamp, 2001). Previous classifications used various methods to classify the vegetation on MI, although all applied the discrete concept of hierarchical plant communities (Gremmen, 1981; Huntley, 1971; Smith & Steenkamp, 2001). The first two classifications of MI were floristic and largely qualitative (Gremmen, 1981; Huntley, 1971). Smith and Steenkamp (2001) then defined 21 habitats in seven habitat complexes based on the main drivers of variation, such as moisture and biotic influence, found with ordination, rather than species occurrence. These previous classifications informed the MI vegetation map that delineated five vegetation units (Smith & Mucina, 2006). We expected to find similar groupings in our data despite using a different methodology since the previous classifications were consistent with each other. However, we found weak substantiation for a floristic community classification with all ISAs having low fidelity, which is a key metric under the Braun‐Blanquette approach. Indeed, if ecologists require discrete communities for management on MI, incorporating the full range of abiotic factors to which species are known to respond, such as wind (Momberg et al., 2021) or soil chemistry (Cramer et al., 2022), may need to be included in the classification.

Our inability to find previously identified communities in the current classification, may be because the previous research did not formally describe the cluster analysis choices in detail or validate the classification (Gremmen, 1981; Smith & Steenkamp, 2001). The justification for a discontinuous view of the vegetation was not described and, as was common in classification research at the time (Lötter et al., 2013), the classification was not methodologically or conceptually specified at the detail necessary to be reproduceable. Therefore, the previously described communities or habitats will likely not be suitable for tracking vegetation change, as they are not objectively reproducible. While expert opinion is invaluable in interpreting classifications, the formal testing of the effectiveness of classifications with various internal and external measures is an essential step that should be reported. Therefore, cluster validation is recommended to improve the quality of the results and increase confidence (Handl et al., 2005). Providing detailed justification for methodological choices in classification research may aid comparisons between classifications and help future researchers in their analytical decision‐making.

A key limitation in the present study is that bryophyte species were not included, unlike Gremmen (1981) who identified all bryophyte species and Smith and Steenkamp (2001) who included some plant guilds of non‐vascular plants. The taxonomic bias towards vascular plant species may have overlooked a substantial part of the vegetation. Nevertheless, regardless of bryophyte species exclusion, vascular plant species were still expected to indicate previously identified communities, as most communities had at least one diagnostic vascular plant species or guild (Gremmen, 1981; Smith & Steenkamp, 2001) and communities dominated by vascular plant species were predicted to become more important (at the expense of bryophytes) in the vegetation as a whole (Smith & Steenkamp, 1990).

Another possible reason for the inability to classify discrete communities, is that the vegetation may have changed rapidly since the previous classifications were formulated, perhaps resulting in species reorganization and novel associations due to climate change (le Roux & McGeoch, 2008a; Raath‐Krüger et al., 2019), as well as an increase in mouse populations due to the eradication of cats (McClelland et al., 2018; Smith & Steenkamp, 1990, 2001; Smith et al., 2001). The previous classifications' fieldwork was conducted at times with much smaller mouse populations, as cats were still present (Gremmen, 1981; Huntley, 1971) and/or recently eradicated (Smith & Steenkamp, 2001), which together with climate change has increased peak mouse densities by 430% from 1979–1980 to 2008–2011 (McClelland et al., 2018). While we cannot definitely establish whether these changes are a cause of the inability to classify discrete communities, sub‐Antarctic vegetation has changed rapidly in recent decades, including changes in plant species distributions (Raath‐Krüger et al., 2019), community reorganization (le Roux & McGeoch, 2008b), changes in phenology (March‐Salas & Pertierra, 2020) and the collapse of an entire ecosystem (Bergstrom et al., 2015). There is thus a possibility that the vegetation has reorganized or become transitional between previously described communities, as was predicted by the authors of previous classifications (Smith et al., 2001; Smith & Steenkamp, 1990, 2001), leading to more continuous vegetation as species expanded their ranges across the island in response to the changes in climate (le Roux & McGeoch, 2008b).

**FIGURE 7:**
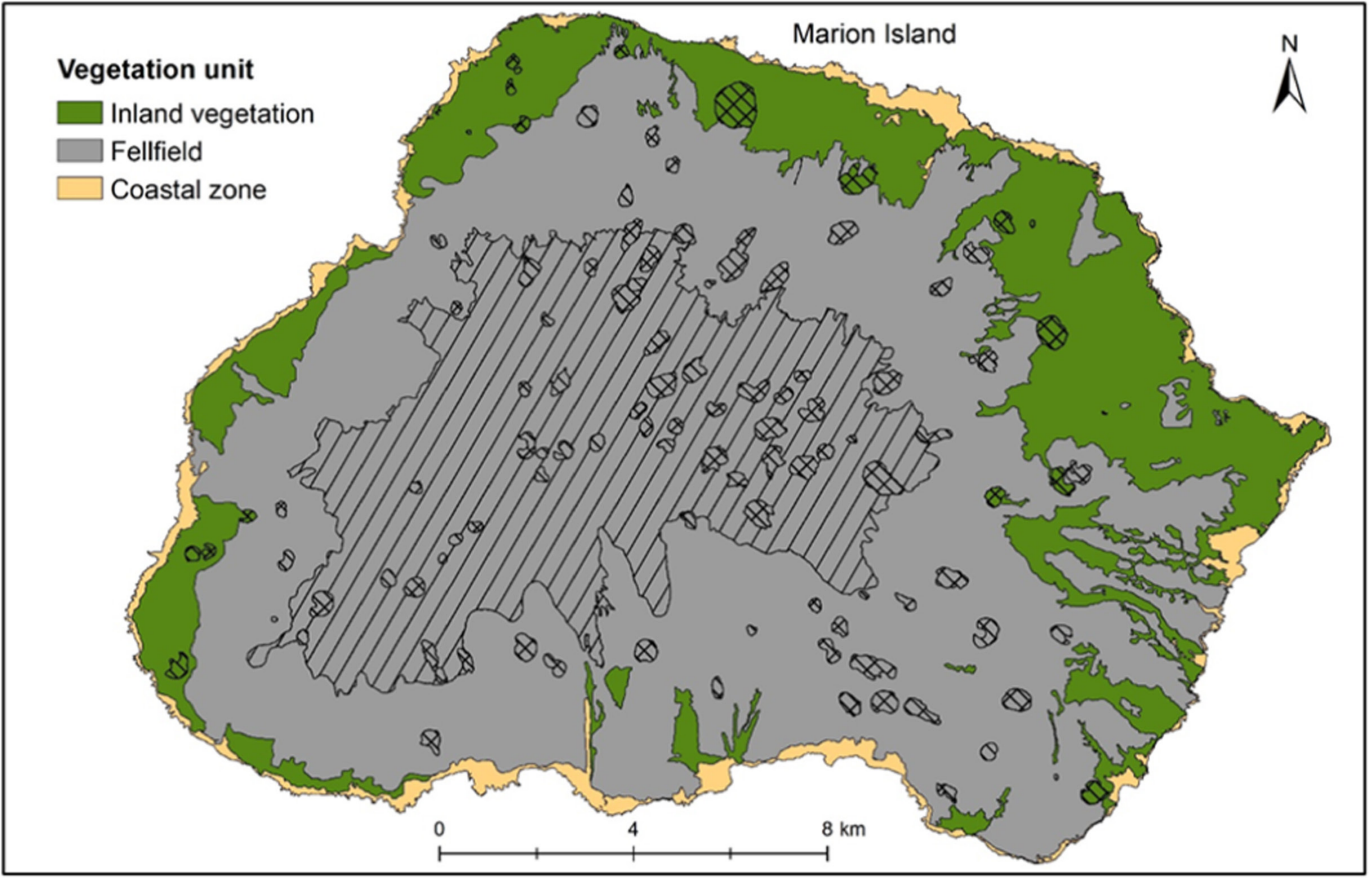
Updated vegetation map of MI showing the three vegetation clusters from the classification in this study. Inland vegetation corresponds to previously mapped “slope” and “mire” complexes. Vegetation previously called “Polar Desert” and “fellfield” were allocated to one cluster called “fellfield” in the current classification due to lack of differentiation by indicator species. The area mapped in the Centre of the map with diagonal lines is near 100% bare rock, which would indicate “Polar Desert”. The coastal zone was clearly separated in the classification by specialist species that only occur along the coast. Cinder cones did not form a vegetation unit but are conspicuous geological features in the landscape with little vegetation and are indicated by crossed lines. The updated map was created with the assumption that the original Smith and Mucina (2006) vegetation map was accurate for their classification.

In this study, the strongest clustering was for two or three clusters. Here, we interpret the three clusters and attempt to relate them to earlier vegetation descriptions that applied a discontinuous view of vegetation variation (Smith & Steenkamp, 2001). From the ISA of three clusters, the first is indicated by the specialist species (see le Roux et al., 2013) on the coast (labeled “Coastal zone” in Figure 7). In the Coastal zone, the biotic nutrient input by seals and seabirds, or salt spray created by rough seas on the high cliffs, increases the nutrient content of soils (Smith & Steenkamp, 2001) and thus creates conditions for species with narrow ecological amplitude to occur. *Crassula moschata* for instance, only occurs where there is high salt spray and thrive in coastal areas where many generalist species cannot (le Roux & McGeoch, 2008b). *Poa annua*, *L. plumosa*, *P. cookii*, *C. antartica* and *M. polymorpha* also occur in the coastal zone in areas with biotic nutrient input (Smith & Steenkamp, 2001). The three most widespread alien plant species are also common here (le Roux et al., 2013). Similarly, in previous classifications, the coastal vegetation was very strongly distinguished as the cluster that differed from all other vegetation (Gremmen, 1981; Smith & Steenkamp, 2001). The next cluster (labeled “Inland vegetation” in Figure 7), is only indicated here by the fern *A. penna‐marina* which is widespread and abundant across the lowlands of the island and occurs occasionally at higher altitudes. It is the dominant species on inland slopes and could be related to the “Slope” complex of previous classifications (Smith & Steenkamp, 2001). The third cluster (labeled “Fellfield” in Figure 7) includes sites with low vegetation cover (i.e., high rock cover), as the only indicators were lichens and *N. crassior* which is a small fern that grows between rock crevices. Despite this attempt to identify clusters, interpreting these as discrete units is misleading because there were no strong grounds for this based on floristic composition, because (a) the silhouette widths and Dunn index were low for any number of clusters, (b) fidelity and specificity to optimal clusters were low and (c) the in‐field descriptions of vegetation did not match well with the three‐cluster classification.

The sub‐Antarctic islands have a relatively recent origin (Rudolph et al., 2020). There have been three glaciations in the last 300,000 years on MI, with the last glaciation reaching a maximum extent around 34,500 years ago, and no evidence of glaciation during the Holocene (Rudolph et al., 2020). Biological refugia, which allowed species to persist, most likely occurred in low lying areas (Rudolph et al., 2020). MI's age and glaciation history, coupled with extreme isolation from continental species pools, has thus resulted in a taxonomically and functionally depauperate system (Smith & Mucina, 2006). Environments with low species richness and turnover do not conform to a methodology that relies on diagnostic species. This is because the vegetation on MI has a high percentage of shared species across the island, and their occurrence does not differentiate between sites, except perhaps along the coast. In older environments (such as Fynbos vegetation in South Africa) that have been stable for millennia and that have contained continental refugia during periods of glaciation (Verboom et al., 2009), community‐level associations may be more apparent, and thus changes more easily monitored.

No comprehensive studies have been published that differentiate criteria for community versus continuum approaches, or under which conditions either are appropriate (Austin, 2013). However, our results align more with the continuum theory where vegetation is viewed as the outcome of individualistic species responses to their environment and to each other (Curtis & McIntosh, 1951; Palmer & White, 1994). Austin (2013) suggested that the continuum concept is preferred in vegetation‐environment investigation. Since the vegetation on MI is closely coupled with the harsh abiotic conditions (Cramer et al., 2022; le Roux & McGeoch, 2008c), a continuum view may better represent the vegetation variation. Indeed, species on MI do respond independently to abiotic conditions (Cramer et al., 2022; Momberg et al., 2021), biotic interactions (Raath‐Krüger et al., 2019) and disturbance (Phiri et al., 2009). The individual responses may vary along gradients, such as the change in vegetation structure along an elevation gradient (le Roux & McGeoch, 2008c). For example, *A. selago*, is a keystone generalist cushion plant species that occurs at low and high elevations, but at different densities (i.e., the structure differs; Phiri et al., 2009). At high elevations, in low densities, *A. selago* facilitates other generalist species that cannot necessarily survive without the protection of cushion plants (Raath‐Krüger et al., 2019). Therefore, while species distributions may overlap at high and low elevations, each responds differently to abiotic conditions and biotic interactions (le Roux & McGeoch, 2008b). The recent rapid change in climate on MI has also altered the distribution of (Raath‐Krüger et al., 2019) and relationships between vascular species with some ranges expanding and others retracting (le Roux & McGeoch, 2008b). This suggests that individual plant species may respond variably to climate change and biotic disturbance (Cramer et al., 2022; Raath‐Krüger et al., 2019).

Despite the acknowledged difficulty in using species fidelity to classify vegetation into communities in cold‐temperate (Gremmen, 1981), species‐poor environments (Landucci et al., 2015), the vegetation on MI continues to be discretely defined at the community‐level, perhaps in order to adhere to the European standard (Braun‐Blanquet, 1932; Mucina et al., 2016). The discrete community concept was originally predominantly used to classify broad‐scale representative stands in environments with sharp compositional boundaries that have high turnover and species fidelity to differentiate communities (Pavão et al., 2019). However, the unit for monitoring vegetation in species‐poor environments should not rely on assemblages of species, but rather individual species, as shown to be more suitable on Macquarie Island (Bricher et al., 2013). Species distribution models (Elith & Leathwick, 2009; Poggiato et al., 2021) could be more promising for differentiating and monitoring vegetation in environments with few vascular plant species that respond individualistically to abiotic conditions, as it predicts species distributions based on their environmental niches (Cramer et al., 2022).

## 5 CONCLUSION

Despite testing a range of clustering and validation methods for MI vegetation, there was no solution that could reliably separate clusters, suggesting that the traditional discrete community view of vegetation may not be appropriate in species‐poor and/or young environments. The marine and terrestrial ecosystems of the sub‐Antarctic have been identified as core areas to understand the rapid climate change that is occurring in the region (Ansorge et al., 2017). In this region, permanent plots to track individual changes in species occurrence and abundance, including bryophytes, across the structural vegetation gradient will likely be more effective to monitor and easily detect real world change than tracking hard to define “plant communities”. Future research should thus focus on the continuous variation in individual species distributions along key environmental gradients, rather than viewing vegetation as discontinuous communities.

## AUTHOR CONTRIBUTIONS

**Stephni van der Merwe:** Conceptualization (lead); data curation (equal); formal analysis (lead); investigation (lead); methodology (lead); project administration (equal); validation (lead); visualization (lead); writing – original draft (lead); writing – review and editing (lead). **Michelle Greve:** Conceptualization (supporting); data curation (equal); funding acquisition (lead); investigation (supporting); methodology (supporting); project administration (lead); supervision (supporting); validation (supporting); writing – review and editing (supporting). **Andrew Luke Skowno:** Conceptualization (supporting); formal analysis (supporting); investigation (supporting); methodology (supporting); supervision (supporting); validation (supporting); writing – review and editing (supporting). **Michael Denis Cramer:** Conceptualization (supporting); formal analysis (supporting); investigation (supporting); methodology (supporting); supervision (lead); validation (supporting); visualization (supporting); writing – review and editing (supporting). **Michael Timm Hoffmann:** Conceptualization (supporting); formal analysis (supporting); investigation (supporting); methodology (supporting); supervision (supporting); validation (supporting); writing – review and editing (supporting).

## Supporting information


Appendix S1

## Data Availability

Floristic plot data used in this manuscript is available on figshare at https://doi.org/10.6084/m9.figshare.21776477.
