# Supplementary material for: Can vegetation be discretely classified in species‐poor environments? Testing plant community concepts for vegetation monitoring on sub‐Antarctic Marion Island
Source: Ecol Evol. 2023 Jan 3;13(1):e9681. doi: 10.1002/ece3.9681 (PMC9811060; doi:10.1002/ece3.9681)
Supplement: Supplementary file 1 — Appendix S1 [file ECE3-13-e9681-s001.docx]

**Appendix**


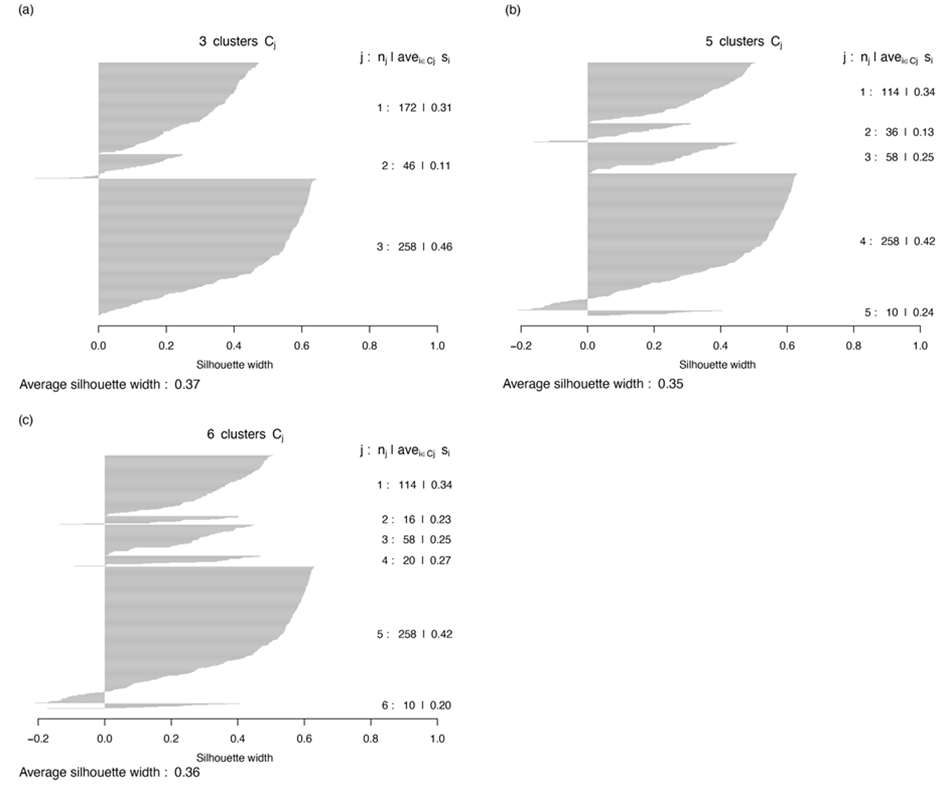


Figure A1. Average silhouette widths calculated for DIANA clustering in a) three groups, b) five groups and c) six groups. The number of clusters were chosen based on the two clustering solutions with highest Average Silhouette Width (ASW) and Dunn statistic, as well as the previously mapped five groups. Each grey horizontal line represents the Silhouette Width of a plot that was allocated to each cluster (j). The number of plots (n_j_; n=476) allocated to each cluster and the ASW for each cluster (ave_ieCiSi_) is shown on the right, as well as the overall average of the entire classification (shown below the graph). Large within-cluster ASW values indicate that plots within a cluster are compositionally similar. A large overall ASW for the entire classification indicates that clusters are well separated and compact. Negative silhouette values indicate plots might have been placed in the incorrect cluster. Ideally, the plots clustered within a group would all have high and similar silhouette widths, i.e. the grey lines would be uniform within a cluster. The overall average would also ideally be high in a well separated and compact grouping of a data set.


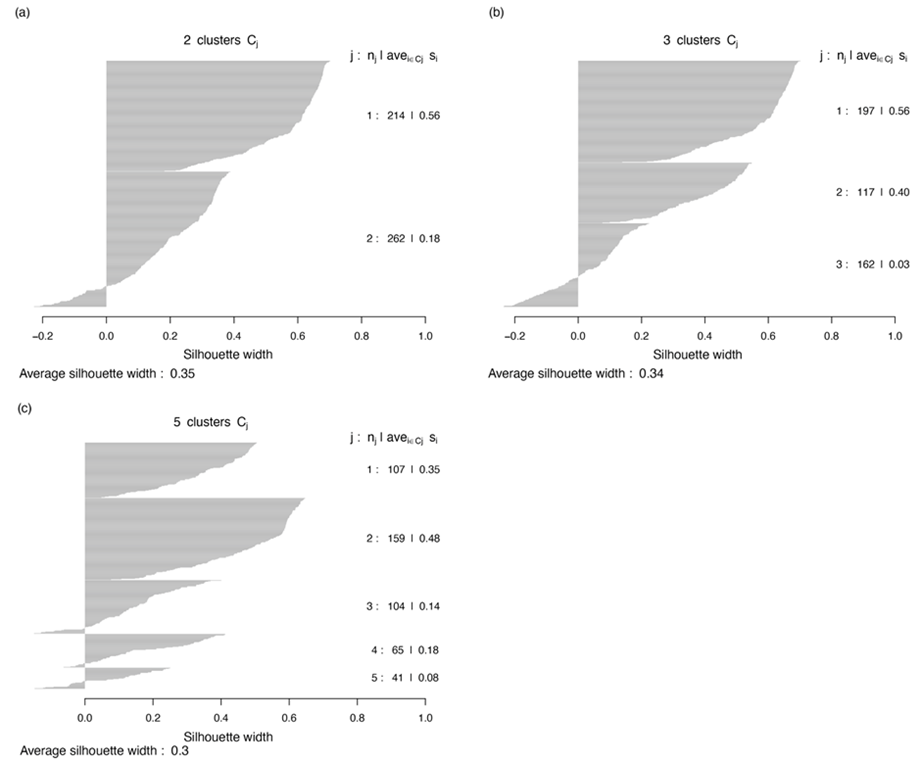


Figure A2. Average silhouette widths calculated for kmeans clustering in a) two, b) three and c) five groups. The number of clusters were chosen based on the two clustering solutions with highest Average Silhouette Width (ASW) and Dunn statistic, as well as the previously mapped five groups. Each grey horizontal line represents the Silhouette Width of a plot that was allocated to each cluster (j). The number of plots (n_j_; n=476) allocated to each cluster and the ASW for each cluster (ave_ieCiSi_) is shown on the right, as well as the overall average of the entire classification (shown below the graph). Large within-cluster ASW values indicate that plots within a cluster are compositionally similar. A large overall ASW for the entire classification indicates that clusters are well separated and compact. Negative silhouette values indicate plots might have been placed in the incorrect cluster. Ideally, the plots clustered within a group would all have high and similar silhouette widths, i.e. the grey lines would be uniform within a cluster. The overall average would also ideally be high in a well separated and compact grouping of a data set.


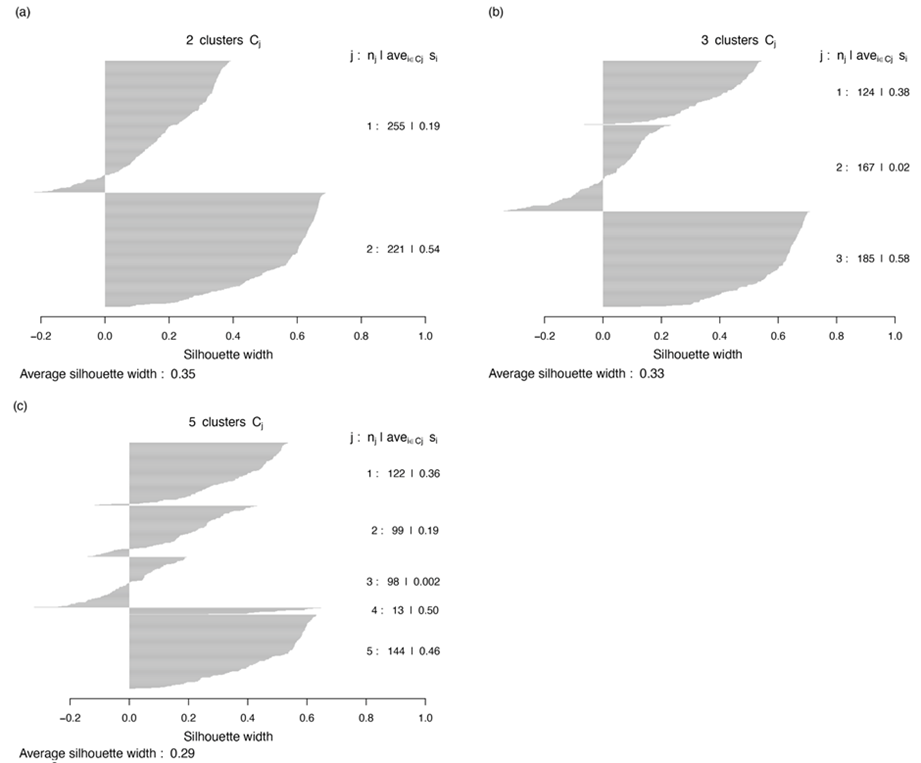


Figure A3. Average silhouette widths calculated for PAM clustering in a) two, b) three and c) five groups. The number of clusters were chosen based on the two clustering solutions with highest Average Silhouette Width (ASW) and Dunn statistic, as well as the previously mapped five groups. Each grey horizontal line represents the Silhouette Width of a plot that was allocated to each cluster (j). The number of plots (n_j_; n=476) allocated to each cluster and the ASW for each cluster (ave_ieCiSi_) is shown on the right, as well as the overall average of the entire classification (shown below the graph). Large within-cluster ASW values indicate that plots within a cluster are compositionally similar. A large overall ASW for the entire classification indicates that clusters are well separated and compact. Negative silhouette values indicate plots might have been placed in the incorrect cluster. Ideally, the plots clustered within a group would all have high and similar silhouette widths, i.e. the grey lines would be uniform within a cluster. The overall average would also ideally be high in a well separated and compact grouping of a data set.


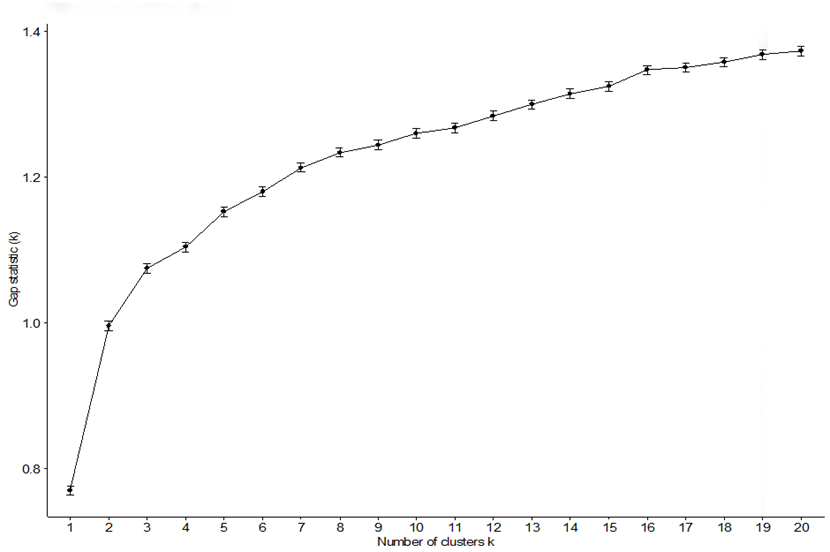


Figure A4. Gap statistic for Ward clustering for 1-20 clusters. The gap statistic compares within-cluster distance to a uniformly distributed null reference distribution with bootstrapping. The optimum cluster number is indicated where the gap curve reaches an inflection point and changes to a higher value.


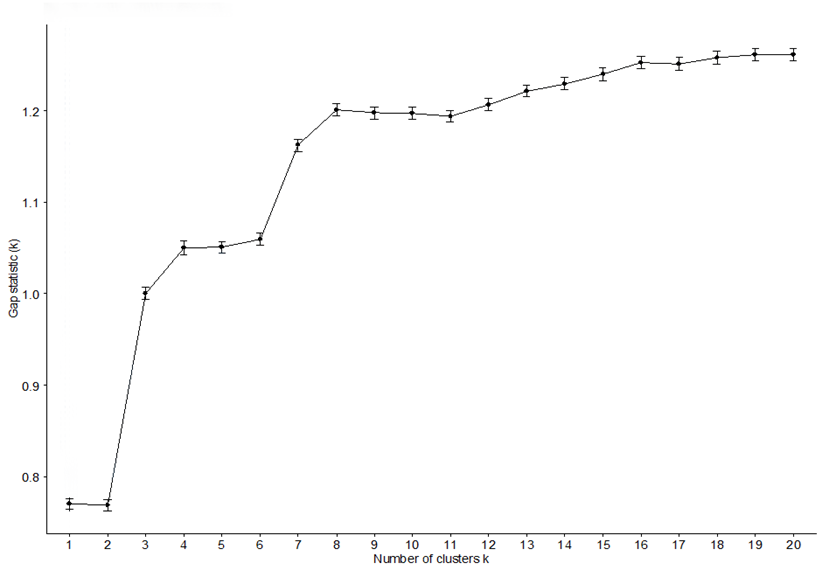


Figure A5. Gap statistic for DIANA clustering for 1-20 clusters. The gap statistic compares within-cluster distance to a uniformly distributed null reference distribution with bootstrapping. The optimum cluster number is indicated where the gap curve reaches an inflection point and changes to a higher value.


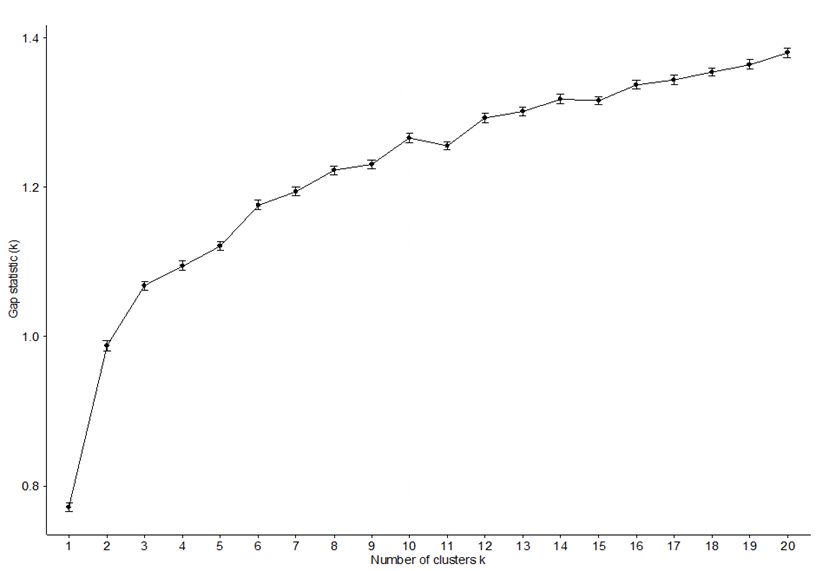


Figure A6. Gap statistic for kmeans clustering for 1-20 clusters. The gap statistic compares within-cluster distance to a uniformly distributed null reference distribution with bootstrapping. The optimum cluster number is indicated where the gap curve reaches an inflection point and changes to a higher value.


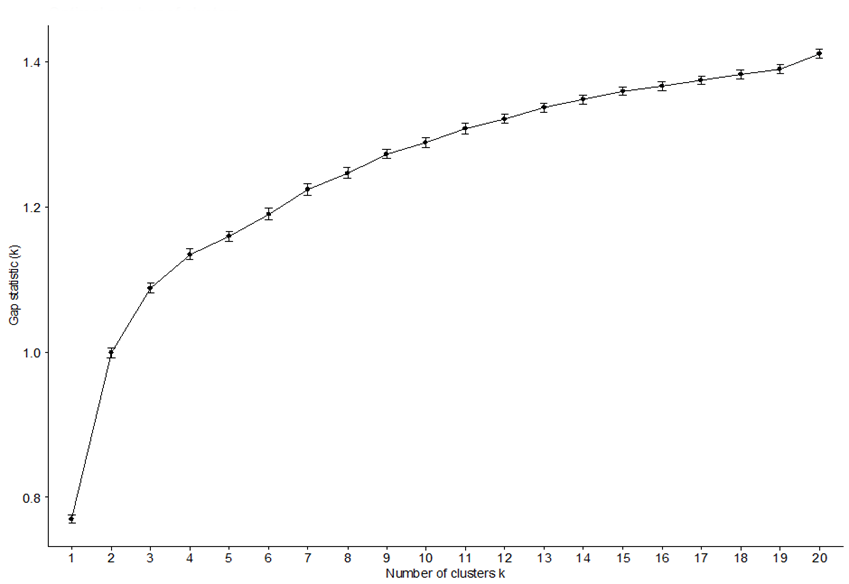


Figure A7. Gap statistic for PAM clustering for 1-20 clusters. The gap statistic compares within-cluster distance to a uniformly distributed null reference distribution with bootstrapping. The optimum cluster number is indicated where the gap curve reaches an inflection point and changes to a higher value.


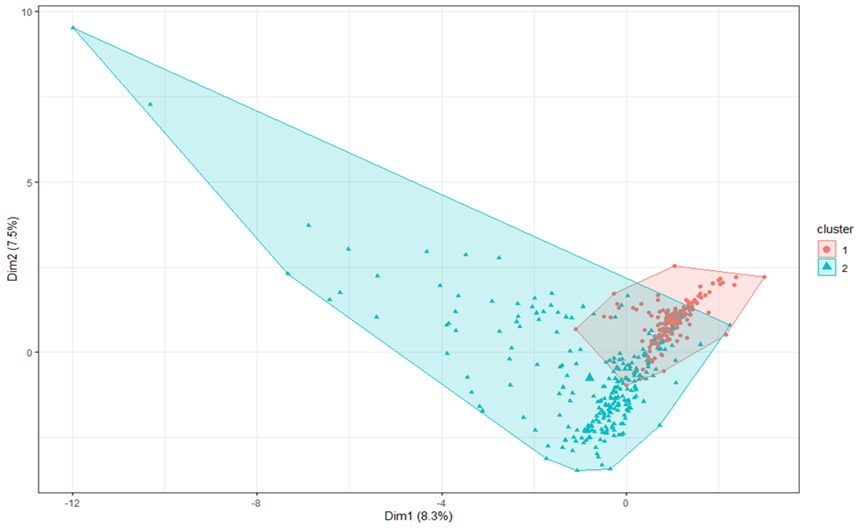


Figure A8. K-means two-cluster solution. Ideally, centroids should be well separated or compact.


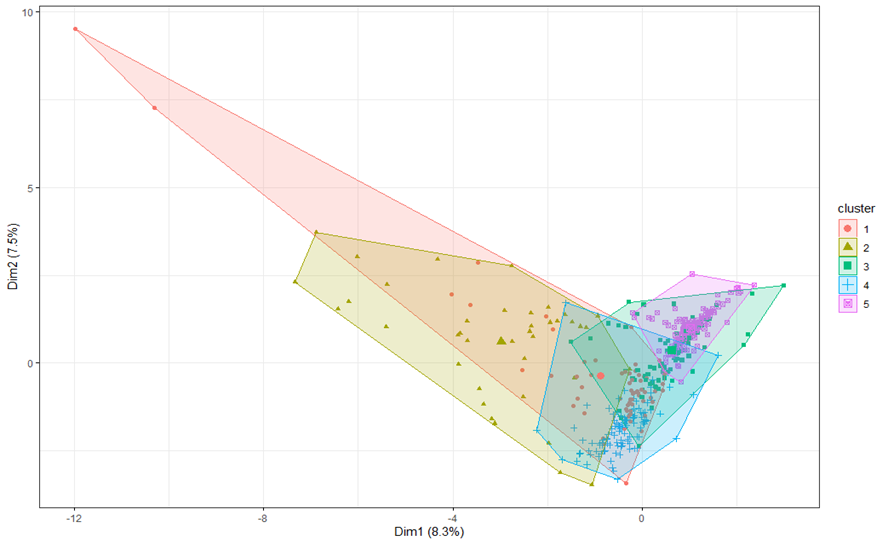


Figure A9. Kmeans five cluster solution. Ideally, centroids should be well separated or compact.


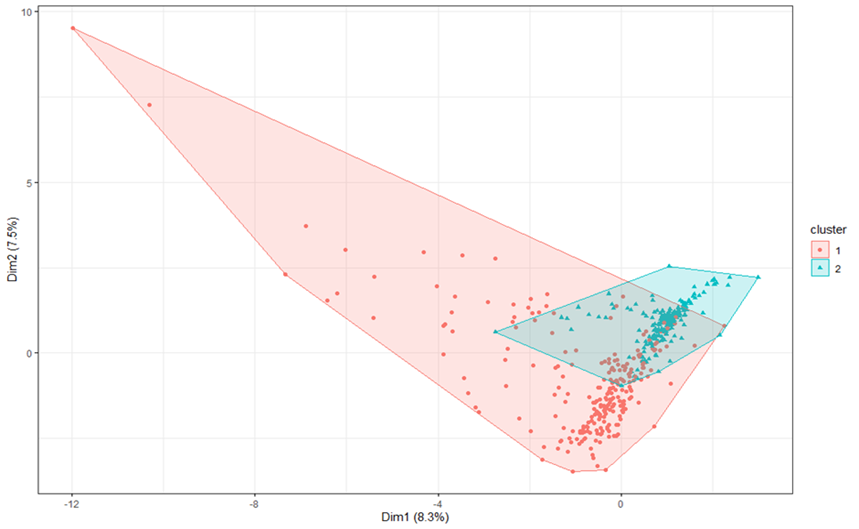


Figure A10. PAM two-cluster solution. Ideally, medoids should be well separated or compact.
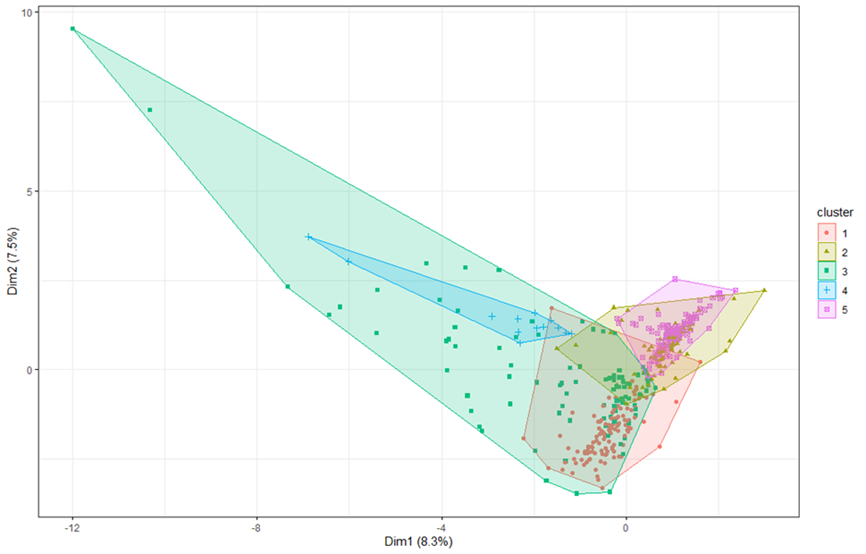


Figure A11. PAM five-cluster solution. Ideally, medoids should be well separated or compact.
